# Hospital Meetings, &c.

**Published:** 1900-04-14

**Authors:** 


### HOSPITAL MEETINGS, &C.

### THE ROYAL EYE HOSPITAL.

The annual general meeting of the Royal Eye Hospital, St. George's Circus, was held on the 28th ult., Professor McHardy, the senior surgeon, presiding. The accounts showed ordinary receipts, £2,283; ordinary expenditure, £2,949; extraordinary receipts (Banquet committee), £2,878, of which £2,100 was invested. There was no extraordinary expenditure. The report stated that during the year the in-patients numbered 591, the average stay being 15·2 days, and the estimated cost of each £2 2s., totalling £1,240. The new out-patients numbered 17,929, the average cost of each of whom was 1s. 11d. The number of attendances was 52,927. The annual dinner on April 21st last, under the presidency of Mr. W. M. Chinnery, was a great success, no less than £4,100 being subscribed. This enabled the Council to open the 14 beds which had been closed so long for want of funds. One of the wards thus opened was called the Stock Exchange Ward, Mr. J. K. Hitchings, the chairman of the Stock Exchange, presiding at the opening on June 6th. Lord Llangattock is to preside at this year's festival.

The report and accounts were adopted, and the council re-elected for the ensuing year. In acknowledging the customary vote of thanks, the Chairman mentioned that a great and continuous strain had been put on their out-patient department, in common with those of other similar institutions, by the action of the London School Board in sending large numbers of children to have their eyesight attended to; and when attendance at the hospital necessitated absence from school, demanding a medical certificate to that effect. This action culminated during the Christmas holidays. In one week alone 800 such children were counted among the new attendances. By extending the staff's hours until as late as seven, eight, and nine p.m., and by precluding them for the time from scientific investigation of rare cases, and abstaining from all attempts at clinical instruction, it was possible to see every new case; but all to whom this first examination showed that serious harm would not result from the delay had to be deferred for future investigation, when the days would be longer, the light better, and the assistants and appliances reinforced. Last month three additional officers were appointed to the out-patient department, at an estimated annual increased cost of at least £200, and in future the department would be open six to eight hours instead of two to four hours every day, as had proved sufficient prior to the action of the School Board. Since Christmas over 3,000 children had applied to them.

Festival Banquet—Characteristic Speech by Professor McHardy.

The festival banquet of the Royal Eye Hospital, Southwark, S.E., took place at the Whitehall Rooms, Hotel Metropole, on Thursday evening, April 5th, under the presidency of the Right Hon. Lord Llangattock. There was a large company of ladies and gentlemen, including Lady Llangattock, Major-General Sir Francis W. de Winton, G.C.M.G., C.B. (treasurer), and Lady de Winton, Sir Francis Astley-Corbett, Bart., and Lady Gertrude Astley-Corbett, Lady Hope, Lady Westbury, Professor M. McHardy (senior hon. surgeon of the hospital), Mr. de F. Pennefather, Mr. W. M. Chinnery (president), Captain and Mrs. Marescaux, Mr. W. A. Toll (chairman of the house committee), Mr. Edwin Easton, &c.

After the usual loyal and patriotic toasts, that of the "Imperial Forces," proposed by Mr. de F. Pennefather, being acknowledged by Major-General Sir Francis de Winton and Major Graham Gordon,

The Chairman gave the toast of the evening, "Success to the Royal Eye Hospital." His words, he said, would be few, but such as they were, they came from a heart warm in sympathy with those who were suffering, warm in his wishes and endeavours to alleviate that suffering as much as possible, for of all the great trials that man had to bear, the greatest, he believed, was the loss of sight. The eye was the most tender and the most wonderful of the many wonderful works of God, and he looked upon this hospital as one of the most wonderful of the many wonderful works of man. He had the pleasure of paying a visit to the hospital on the previous day, and was most kindly received by Professor McHardy. Everything, he found, was perfect. There had been nothing forgotten that could be for the safety, comfort and convenience of the patients. There was only one thing wanting, and that was money. (Laughter.) But had they ever heard of anybody who did not want more money? He never had. (Hear, hear.) The hospital was founded in 1857 for the free treatment of poor persons suffering from eye disease or injury. There was a splendid array of royal patrons; there were acres of vice-presidents—(laughter)—and, what was much more important than all, they had on the medical staff some of the best physicians and surgeons in the world. (Cheers.) From the report for 18[illegible] he saw that over 18,000 new patients had been registered and nearly 53,000 out-patients had been attended to, although the annual income was only about £3,000. The actual value of the medical services given freely amounted to £26,00[illegible]. This was magnificent, and the thanks of the whole country were due to the medical officers for their splendid work. He was bound to say that if he had the misfortune to have his eye taken out he would certainly like to go to Professor McHardy, who would do it in such a happy, cheery manner that he (his Lordship) would rather enjoy the operation. (Laughter.) What a blessing such institutions were; what an opportunity of doing good. To those who had so generously subscribed to the hospital during this unfortunate year their thanks were most cordially due. They had the consolation of knowing that it was more blessed to give than to receive. There was no man, after all, who was happy if he led a selfish life. The happy man was he who tried to make others happy, and liked to join them in all their pleasures and sympathise with them when they were in trouble. Those were the principles which should be engravened in the heart of every man, whatever might be his mode of life. That the hospital might increase in funds, in usefulness, and in its good works was his earnest hope. (Cheers.)

The toast was enthusiastically honoured.

Professor McHardy, who was very enthusiastically received, acknowledged the toast in a characteristic speech, in the course of which he said: It is necessary for me to review the occasion and how it comes about that the Royal Eye Hospital at Southwark has to ask anyone to give it anything. I see from the figures which the noble chairman has given the extraordinary fact that the medical profession, from which I disassociate myself entirely for the moment—(laughter)—are giving us eight to one in comparison with the British public. Now, what is the proportion of the medical profession to the British public? It is one to 800, or one to 1,000. The members of that profession come from all classes. Why are they eight, eight hundred, or even eight thousand times more philanthropic to the sick and suffering than the British public? I was staggered by this question, but I looked it in the face, and it seems to me that the answer is exceedingly simple. The medical profession, seeing what it is possible to do with half-pence, pence, shillings, and paltry sovereigns—(laughter)—and seeing what little they can do, cannot withhold that little. It is the proud privilege of the medical profession to be able to produce a picture with that paint and palette which you have only to recognise it as your privilege to provide. I am here in a new situation altogether; I am the remnant of a beggar. Last Sunday week this festival looked like a terrible failure. Friends said, "Mac, old man, why did you have a dinner in Lent?" I said, "Because I do not want to borrow." They said, "You should have had it later in the season, old man." I said, "Not a bit of it." They said, "You should never have had it in this year of khaki fever, of sick and wounded funds, and of Imperial Yeomanry." This latter remark touched me, for I have lost my senior assistant, Mr. Cargill—(applause)—who has gone out with the Imperial Yeomanry. I do not profess to have any eloquence, but I do believe that, incapable as I am, even after dinner—(laughter)—of making a speech, you will understand me when I ask those of you who have written down one figure to make it two, and those who have given a donation to convert it into an annual subscription. (Laughter and cheers.) This hospital has a few passwords, among them "I's" (eyes), of course—nothing like egotism, and "efficiency" before everything else. Our noble chairman and others in the same position can choose to whom they will go, not to have an eye out, as he has said, but rather to have it saved; but we minister to the poor, who cannot have their choice, and who, for the preservation of the most precious faculty, we desire should have the same means, appliances, and advantages as those which our noble chairman has at his command. (Hear, hear.) At the hospital, I will say without fear of contradiction, the poorest, the hungriest, and dirtiest, night and day, can have every assistance that is likely to preserve for him that most precious of all functions, good eyesight. (Cheers.) During the thirty years it has been my privilege to work at the hospital I have seen the new out-patients grow from something like 3,600 in the year to, roughly, 18,000. I believe the hospital is perfect, and I attribute that entirely to the personal sagacity of the council. They appointed a rebuilding sub-committee, with the full powers of the council, and that sub-committee did its work to my entire satisfaction. (Laughter.) It worked heartily, continuously, and effectively. When that hospital was built it was unique in that it was a model for all the world to copy, and, I hope, improve upon. It was opened out of debt. I found that the church had the knack of going into debt, and I determined to go on another tack—(laughter)—and start by being unique. I started with the hospital out of debt, and we have always kept out of debt. I assure you that while I have the power I shall always have the will to spend; it is your fault if I have not the power. There shall be no hoarding here, except such hoarding as may be necessary while we extend the premises. We will not put our money in a napkin and wrap it up. We will apply it so that it pays a percentage unknown to the avaricious mind. (Cheers.) You cannot conceive personally what a return we get for every sixpence spent here. Upon my soul and honour, since I have realised what the £2,950 spent at the Royal Eye Hospital last year has done, I am ashamed to spend a single sixpence on anything else. (Laughter and cheers.) That is the feeling which should imbue every one of you who believes one-tenth part of what I say, and really I have not the power of imagination. (Renewed laughter and cheers.) That hospital, if you will support it a little more, will do a great deal more. Here is a quotation from Rudyard Kipling; it refers to an individual called "Bobs," but it is applicable also to the Royal Eye Hospital:—

> "He's little, but he's wise;
> He's a terror for his size,
> And he does not advertise."

The hospital spends not a sixpence for advertisements. "By their fruits ye shall know them." The Professor concluded: The collection will now be taken. I hope it will be a good one. (Cheers.)

The Secretary subsequently announced that the total fund of the festival was, roughly speaking, £1,050. The chairman's list was about £450, including £100 from himself; Professor McHardy's list was about £350; and the ladies' list £150. (Cheers.)

Mr. Matthew Clark gave "The Ladies," to which Mr. C. P. Johnson responded; and Professor McHardy, acknowledging the toast of "The Staff of the Institution (proposed by Mr. Gilbert Purvis) expressed regret at the absence through illness of Dr. Collins, the honorary surgeon.

Mr. Chinnery proposed "The Health of the Chairman," and the proceedings terminated.

### NATIONAL HOSPITAL FOR THE PARALYSED AND EPILEPTIC.

The forty-first annual meeting of the National Hospital for the Paralysed and Epileptic, Queen Square, Bloomsbury, was held on the 5th inst, the Chairman of the Board of Management, Colonel Porter, presiding. The report presented deplored the loss the hospital had sustained on the death of its president, the Duke of Westminster, who had been connected with it for many years; and went on to appeal for greater support with a view to the enlargement of the hospital in view of the widespread demands for its assistance. They had only 200 beds, the board stated, and the average stay of patients was three times as long as that of those in a general hospital. The total number of in-patients during the past year was 1,032, and the average of beds occupied 176. The cost of each occupied bed was £76 19s. 5d. The number of out-patients treated advanced from 5,969 in 1898 to 6,170 in 1899. The attendances, however, fell from 37,677 to 37,065. Arrangements had been sanctioned by which out-patients who were able contributed towards the cost of medicine supplied to them. The results had been satisfactory. About three-fifths of the whole number of patients were now willing contributors, over £800 per annum being received from this source. When the leases of adjoining houses, the property of the hospital, fell in, which would give them the necessary ground, the board proposed to take steps which would allow of an urgently-needed re-arrangement of the out-patient department, and provide accommodation for an inquiry officer. This work was included among the items to be met by the "Commemoration Fund" of £10,000, towards the completion of which £2,721 was still required. The ordinary income for the year was £14,482, and the ordinary expenditure £15,047. The extraordinary income (legacies) was £4,168, and the extraordinary expenditure £139. Among the legacies which fell in was that of Mr. Dymoke, the first ever bequeathed to the charity. The will was dated 1861, but, being reversionary, the legacy had only become payable during the past year. The value of the convalescent home at Finchley had again been amply demonstrated, the admissions totalling 166, the patients in most cases being ineligible for any other convalescent home. Since the accounts had been made up £600 had been received from Mrs. Edward Silva for the foundation of a pension of £20 in memory of her late husband, whose death the board recorded with deep sorrow and a full appreciation of his services to the institution. Regret was also expressed at the death of Sir Charles Hall, who presided at the festival dinner last May; of Mr. Wm. Adams, long a member of the staff and for the past nine years its consulting surgeon; and of Miss M. L. Parker, who was a zealous member of the Ladies' Samaritan Society, and whose connexion with the charity dated almost from the beginning. The resignation was recorded, owing to considerations of health, of Miss Rachel F. Tweed of the post of lady superintendent. The board expressed their sense of the good work done by Miss Tweed during the twelve years of her service. Miss Eleanor C. Vernet has been elected to fill the vacancy.

The report and accounts were adopted, the retiring members of the board were re-elected, and the proceedings terminated with the customary votes of thanks.

### SAMARITAN FREE HOSPITAL.

The annual meeting of the Samaritan Free Hospital for Women and Children, Marylebone Road, N.W., was held at the hospital on April 3rd at five p.m., when the fifty-third report was presented and accepted. Mr. J. R. Diggle, M.A , J.P., chairman of the committee of management, presided. Colonel Watson moved, and the Rev. J. Hutchons seconded, without comment, the adoption of the report. The Chairman drew attention to the rearrangement of the financial statement, by which the approximate proportion of the expenditure caused by the out-patient department was indicated. The resolution was then put to the meeting and carried. The Rev. A. W. Oxford, M.A., M.D., moved the re-election of the committee of management, which Mr. Wheen seconded. The Chairman moved the election as vice-presidents of Mr. Nash, Dr. Rogers, and Dr. Day, in accordance with the recommendation of the committee. Colonel Watson seconded the motion, which was accepted. The Chairman next brought forward the revised rules for the management of the hospital, proofs of which were already before the governors. He explained that the matter was chiefly one of a more convenient rearrangement of the old regulations under their appropriate headings; whilst the verbal alterations effected the literature more than the sense. Dr. Oxford moved their adoption, and Mr. Wheen seconded the motion. Colonel Watson moved that the meeting approve the action of the committee of management in granting their late secretary, Mr. Scudamore, a pension of £144 18s. per annum. He pointed out that Mr. Scudamore's health was permanently and irretrievably broken down. Mr. Hutchons seconded the resolution, which was approved. A vote of thanks to the chairman terminated the proceedings. The report declared that the number of in-patients for 1899 was 517, against 532 for the previous year. The cause of the smaller number was the greater severity of the cases admitted. The number of out-patients also showed a decrease occasioned by the greater strictness in selecting only suitable cases. The figures are 7,430 for 1899, against 8,093 in 1898. Attention is drawn also to the wide radius from which patients are admitted. Two hundred and twelve cases came from 27 English, two from Welsh, and one from Scotch counties. The year has been a difficult one financially. The receipts show diminution from legacies and subscriptions, but the grants from the Prince of Wales's, the Saturday, and the Sunday Hospital Funds are satisfactorily larger, whilst the expenses of administration have been reduced from £856 in 1898 to £749 last year. Mr. W. Grintrip King, assistant secretary, has been appointed secretary in place of Mr. Scudamore, resigned.

